# Invasive Aspergillosis after Pandemic (H1N1) 2009

**DOI:** 10.3201/eid1606.100165

**Published:** 2010-06

**Authors:** Asma Lat, Nahid Bhadelia, Benjamin Miko, E. Yoko Furuya, George R. Thompson

**Affiliations:** New York–Presbyterian Hospital, New York, New York, USA (A. Lat, N. Bhadelia, B. Miko, E. Yoko Furuya); University of California, Davis, Sacramento, California, USA (G.R. Thompson III)

**Keywords:** Invasive aspergillosis, pandemic (H1N1) 2009, fungi, viruses, CME, dispatch

## Abstract

We report 2 patients with invasive aspergillosis after infection with pandemic (H1N1) 2009. Influenza viruses are known to cause immunologic defects and impair ciliary clearance. These defects, combined with high-dose corticosteroids prescribed during influenza-associated adult respiratory distress syndrome, may be novel risk factors predisposing otherwise immunocompetent patients to invasive aspergillosis.

## CME ACTIVITY

Medscape, LLC is pleased to provide online continuing medical education (CME) for this journal article, allowing clinicians the opportunity to earn CME credit. This activity has been planned and implemented in accordance with the Essential Areas and policies of the Accreditation Council for Continuing Medical Education through the joint sponsorship of Medscape, LLC and Emerging Infectious Diseases. Medscape, LLC is accredited by the ACCME to provide continuing medical education for physicians. Medscape, LLC designates this educational activity for a maximum of 0.25 *AMA PRA Category 1 Credits*™. Physicians should only claim credit commensurate with the extent of their participation in the activity. All other clinicians completing this activity will be issued a certificate of participation. To participate in this journal CME activity: (1) review the learning objectives and author disclosures; (2) study the education content; (3) take the post-test and/or complete the evaluation at **www.medscapecme.com/journal/eid**; (4) view/print certificate.

## Learning Objectives

Upon completion of this activity, participants will be able to:

- Describe diagnostic and management strategies for invasive aspergillosis following influenza infection.
- Identify historical outcomes of influenza-associated invasive aspergillosis.

## Editor

**Thomas J. Gryczan**, Copyeditor, *Emerging Infectious Diseases. Disclosure: Thomas J. Gryczan has disclosed no relevant financial relationships.*

## CME Author

**Charles P. Vega, MD**, Associate Professor; Residency Director, Department of Family Medicine, University of California, Irvine. *Disclosure: Charles P. Vega, MD, has disclosed no relevant financial relationships.*

## AUTHORS

*Disclosures:* ***Asma Lat, PharmD; Nahid Bhadelia, MD; Benjamin Miko, MD****; and* ***E. Yoko Furuya, MD, MSc****, have disclosed no relevant financial relationships.* ***George R. Thompson III, MD****, has disclosed the following relevant financial relationships: served as an advisor or consultant for Basilea Pharmaceutica Ltd.; served as a speaker or member of a speakers bureau for Merck & Co., Inc.; received grants for clinical research from Pfizer Inc.*

## Earning CME Credit

To obtain credit, you should first read the journal article. After reading the article, you should be able to answer the following, related, multiple-choice questions. To complete the questions and earn continuing medical education (CME) credit, please go to **www.medscapecme.com/journal/eid**. Credit cannot be obtained for tests completed on paper, although you may use the worksheet below to keep a record of your answers. You must be a registered user on Medscape.com. If you are not registered on Medscape.com, please click on the New Users: Free Registration link on the left hand side of the website to register. Only one answer is correct for each question. Once you successfully answer all post-test questions you will be able to view and/or print your certificate. For questions regarding the content of this activity, contact the accredited provider, CME@medscape.net. For technical assistance, contact CME@webmd.net. American Medical Association’s Physician’s Recognition Award (AMA PRA) credits are accepted in the US as evidence of participation in CME activities. For further information on this award, please refer to http://www.ama-assn.org/ama/pub/category/2922.html. The AMA has determined that physicians not licensed in the US who participate in this CME activity are eligible for *AMA PRA Category 1 Credits*™. Through agreements that the AMA has made with agencies in some countries, AMA PRA credit is acceptable as evidence of participation in CME activities. If you are not licensed in the US and want to obtain an AMA PRA CME credit, please complete the questions online, print the certificate and present it to your national medical association.

### Article Title: Invasive Aspergillosis after Pandemic (H1N1) 2009

## CME Questions

1. Mr. Washington is a 35-year-old patient with a 2-day history of high fever, malaise, and cough. His son was diagnosed with infection with H1N1 influenza last week. None of the family was vaccinated against H1N1 influenza. He receives supportive care only for his symptoms. However, he presents to the emergency department the following week. He has experienced significant shortness of breath, and a chest x-ray reveals bilateral infiltrates. Mr. Washington is started on antibiotics but decompensates and develops ARDS.
  Which of the following diagnostic strategies in this case is most appropriate if invasive aspergillosis (IA) is suspected?
  A. The potential influenza infection does not influence the risk for IA in this immunocompetent patient, and further assessment for IA is not indicated
  B. Bronchoscopy and culture for aspergillosis
  C. Bronchoscopy with direct smear for aspergillosis
  D. Serum galactomannan assay
2. Mr. Washington is diagnosed with IA. On the basis of this activity, which of the following strategies should be considered?
  A. Aggressive early treatment with antiviral medications
  B. Rapid diagnosis of fungal infection after the initial bronchoscopy
  C. Treatment with corticosteroids
  D. Initial treatment with voriconazole

### Activity Evaluation

**Table:** 

**1. The activity supported the learning objectives.**				
Strongly Disagree				Strongly Agree
1	2	3	4	5
**2. The material was organized clearly for learning to occur.**				
Strongly Disagree				Strongly Agree
1	2	3	4	5
**3. The content learned from this activity will impact my practice.**				
Strongly Disagree				Strongly Agree
1	2	3	4	5
**4. The activity was presented objectively and free of commercial bias.**				
Strongly Disagree				Strongly Agree
1	2	3	4	5

## Invasive Aspergillosis after Pandemic (H1N1) 2009

Invasive aspergillosis has emerged as a major cause of life-threatening infections in immunocompromised patients. Patients with prolonged neutropenia, inherited immunodeficiency, or those receiving immunosuppressive agents are at risk for infection (*1*). Recent evidence has identified other populations at risk for invasive aspergillosis, including those with chronic obstructive pulmonary disease (COPD) and nontransplant patients in intensive care units (ICUs) (*2**,**3*). We recently treated 2 immunocompetent patients who had invasive aspergillosis after infection with pandemic (H1N1) 2009 and are aware of similar cases at other centers (*4*).

Influenza strains have been reported to cause cell-mediated defects, disruption of normal ciliary clearance (*5*), and leukopenia (*6*). These abnormalities and use of high-dose corticosteroids during treatment for influenza-associated acute respiratory distress syndrome (ARDS) may form a unique group of risk factors for invasive aspergillosis.

## The Patients

Patient 1 was a 28-year-old man (college student) with no unusual medical history (body mass index 18 kg/m^2^) who was hospitalized after having shortness of breath for 1–1.5 weeks. Upon admission, he required intubation for respiratory failure. A chest radiograph showed multilobar pneumonia. His condition was complicated by pneumothoraces and bronchopleural fistula formation secondary to barotrauma during mechanical ventilation. A nasopharyngeal influenza swab specimen obtained at admission was positive for influenza A by PCR and was confirmed as pandemic (H1N1) 2009 by the New York State Health Department. Results of bronchoscopic evaluation for copathogens were negative. The patient was not treated with antivirus medication during his hospitalization because the diagnosis was made outside the optimal treatment time frame.

He received high-dose methylprednisolone (1 mg/kg/day) for ARDS for 28 days and later underwent bronchoscopy because of poor clinical improvement. Necrosis of the airway wall and cartilage, with extensive hyphae, was found and tissue cultures showed *Aspergillus fumigatus*. Intravenous voriconazole was initiated (6 mg/kg every 12 h [2 doses], then 4 mg/kg every 12 h for 24 days), but his clinical condition deteriorated; micafungin (100 mg/d) was added and continued for 14 days until he was transferred to another hospital on day 52.

On admission, tests showed lymphopenia (700 cells/mm^3^) and renal and respiratory failure. *A*. *fumigatus* was isolated from sputum, bronchoscopy, and pleural fluid cultures. A computed tomographic scan of the chest showed multifocal pneumonia. He was initially treated with amphotericin B lipid complex, cefepime, metronidazole, tobramycin, and vancomycin. The patient became hemodynamically unstable and required multiple vasopressors. On day 70, he had cardiopulmonary arrest and died. An autopsy was not performed.

Patient 2 was a 51-year-old man (office worker) with no unusual medical history (body mass index 24.5 kg/m^2^) who was hospitalized for fatigue and fever (<104°F). A chest radiograph showed bilateral infiltrates. Laboratory tests showed a leukocyte count of 1,500 cells/mm^3^ and a thrombocyte count of 65,000 cells/mm^3^. A bone marrow biopsy specimen was negative for malignancy. A computed tomographic scan of the chest showed diffuse alveolar consolidation. Broad-spectrum antimicrobial drugs (vancomycin, aztreonam, azithromycin, and fluconazole) were given but the patient’s respiratory status rapidly deteriorated and he was intubated on hospital day 2. Bronchoscopy showed alveolar hemorrhage, and he underwent video-assisted thoracoscopy and right lung wedge resection.

Virus cultures were positive for influenza A and verified as pandemic (H1N1) 2009 by the New York State Health Department. Antiviral treatment was not started because the diagnosis was made >48 hours after the onset of symptoms. A lung biopsy specimen showed widespread alveolar hemorrhage without fungal elements. Development of ARDS prompted administration of methylprednisolone (1 mg/kg/day for 3 days); his fever was quickly reduced. Once the methylprednisolone dose was tapered, fever recurred. Bronchoscopy on day 12 showed spontaneous bleeding from the right middle lobe and multiple blood clots. Pathologic examination identified fungal hyphae and *A. fumigatus* grew in culture. Treatment with intravenous voriconazole (6 mg/kg every 12 h [2 doses], followed by 4 mg/kg every 12 h) was initiated and continued for 3 days until he was transferred to another hospital on day 16.

At the new hospital, broad-spectrum antimicrobial therapy (linezolid, cefepime, tobramycin, oseltamivir, and voriconazole) was initiated. The patient showed decompensation over the next 72 h; his family withdrew care on day 21, and the patient died later that day. An autopsy showed severe pulmonary congestion, hemorrhage, and acute necrotizing bronchopneumonia. Several fungal abscesses consistent with *Aspergillus* spp. were identified in the lung, thyroid gland, and liver.

## Conclusions

The number of patients at risk for invasive aspergillosis continues to increase. Recently, patients with COPD who are receiving long-term corticosteroids and immunocompetent ICU patients have been identified as nontraditional hosts at risk for invasive aspergillosis. Mortality rates in these groups are high, ≈95% in COPD patients (*2*) and 80% in ICU patients (*3*). However, infection with influenza and other respiratory viruses may pose a similar risk for invasive aspergillosis. Despite these high mortality rates, this association remains largely unnoticed (*7*–*10*). Thus, *Aspergillus* spp. observed in bronchoscopically obtained cultures from ICU patients diagnosed with pandemic (H1N1) 2009 may be overlooked as a contaminant despite their potential to cause invasive disease.

Infection with influenza virus is known to cause cell-mediated defects, disruption of normal ciliary clearance after infection (*5*), and leukopenia (*6*). These symptoms may predispose patients for invasive fungal disease. Additionally, ARDS (*11*) and immunodysregulation (*12*) may develop in patients with pandemic (H1N1) 2009. Severe structural lung disease apparent in this syndrome may also impair ciliary clearance, further predisposing these patients to invasive infections. Data suggest a potential benefit of corticosteroids in treating ARDS patients (*13*). Although potentially life-saving, high-dose corticosteroids, combined with structural and immunologic abnormalities observed in patients with pandemic (H1N1) 2009, may predispose patients to invasive aspergillosis. Development of this disease after influenza may be a rare complication, but infection with pandemic (H1N1) 2009 is widespread, thus placing many patients at risk for invasive aspergillosis.

Previous studies of influenza-associated aspergillosis have reported mortality rates of 100% (*7*–*10*). However, most of these reports predate routine use of noninvasive markers of invasive appergillosis or availability of voriconazole. Previous reports were published before the availability of oseltamivir, and specific antivirus therapy has been shown to decrease the incidence of influenza-associated complications (*14*,*15*). Early treatment with oseltamivir may have prevented complications seen in our patients.

Although intubated ICU patients commonly undergo bronchosopy, lack of a positive culture or direct smear result does not rule out a diagnosis of invasive aspergillosis (*3*). Moreover, although radiographic imaging may suggest aspergillosis, invasive diagnostic tests may be impractical when patients are hemodynamically unstable or have severe hypotoxicity, thrombocytopenia, or advanced coagulation deficits (*1*). Before serum testing for galactomannan, these patients would have satisfied criteria only for possible invasive aspergillosis and appropriate treatment could have been withheld. However, assays for detection of serum galactomannan or 1–3-β-D glucan and compatible imaging studies can aid in the diagnosis of probable invasive aspergillosis and thus the initiation of appropriate antifungal therapy. With increased awareness of invasive aspergillosis in nontraditional hosts, high mortality rates in patients with this disease can be avoided.

In conclusion, we report 2 patients with invasive aspergillosis after infection with pandemic (H1N1) 2009. Development of ARDS, structural lung disease, high doses of corticosteroids, and T-cell defects during infection with influenza viruses may be responsible for an emerging group of patients at high risk for invasive aspergillosis. Early diagnostic and treatment strategies should be used for these patients, and multicenter studies are needed to better define incidence and outcomes.
